# Cervical giant Nabothian cysts in a woman with primary infertility: a case report

**DOI:** 10.1186/s13256-025-05393-x

**Published:** 2025-07-09

**Authors:** Sedigheh Hosseini, Parisa Taherzadeh Boroujeni, Nazanin Hajizadeh, Mahsa Kazemi, Leila Majdi, Hamidreza Mosleh

**Affiliations:** 1 Department of Obstetrics and Gynecology, School of Medicine, Shahid Beheshti University of Medical Sciences, Tehran, Iran; 2 Department of Biology and Anatomical Sciences, School of Medicine, Shahid Beheshti University of Medical Sciences, Tehran, Iran; 3 Preventative Gynecology Research Center, Taleghani Hospital Clinical Research Development Unit, School of Medicine, Shahid Beheshti University of Medical Sciences, Arabi St., Student Blvd., Valenjak, Tehran, 19857-11151 Iran; 4 Department of Cell Biology and Anatomical Science, Faculty of Medicine, Shahid Beheshti University of Medical Science, Tehran, Iran

**Keywords:** Nabothian cyst, Cervical cyst, Infertility, Assisted reproductive technology, Case report

## Abstract

**Background:**

Nabothian cysts are benign cervical lesions commonly observed in women of reproductive age, typically ranging from 2 to 10 mm in diameter and often asymptomatic. These cysts arise from the obstruction of cervical mucous glands, a phenomenon frequently linked to childbirth, minor trauma, or chronic cervicitis. While small Nabothian cysts are usually incidental findings, giant Nabothian cysts—those exceeding 4 cm—are rare and can present diagnostic and therapeutic challenges. Their size and appearance may mimic malignant entities such as adenoma malignum, necessitating advanced imaging and histopathological evaluation. Although their association with infertility remains controversial, some evidence suggests that large cysts might interfere with fertility by obstructing the cervical canal or altering mucus production, which is critical for sperm transport. This report examines a rare case of giant Nabothian cysts in the context of assisted reproductive technology, highlighting their management and potential implications for infertility treatment.

**Case presentation:**

A 41-year-old Iranian woman with a 2-year history of primary infertility presented to our clinic. She reported regular menstrual cycles and no symptoms such as pelvic pain or abnormal discharge. During her infertility evaluation, transvaginal ultrasonography identified multiple large cervical cysts (20–45 mm) obstructing the cervical os. Subsequent magnetic resonance imaging and biopsy confirmed these as benign Nabothian cysts. Her partner’s semen analysis revealed severe teratozoospermia, prompting the use of intracytoplasmic sperm injection. During oocyte retrieval, the cysts were aspirated to prevent potential complications during embryo transfer. Two high-quality embryos were transferred, but the cycle did not result in pregnancy. Cytological analysis of the aspirated fluid reaffirmed the benign nature of the cysts.

**Conclusion:**

This case demonstrates that giant Nabothian cysts can be safely aspirated during an assisted reproductive technology cycle, potentially improving procedural outcomes. However, the lack of pregnancy suggests that, while cyst management may address mechanical barriers, it does not guarantee success in multifactorial infertility cases. Further studies are needed to elucidate the role of Nabothian cysts in infertility and refine their management in assisted reproductive technology settings.

## Introduction

Nabothian cysts, also termed retention or mucinous cysts, are a common finding in women of reproductive age, with a reported prevalence of up to 12% in routine gynecological examinations [1]. These cysts form when the cervical mucous glands, responsible for producing mucus to facilitate sperm transport, become obstructed. Common causes of this obstruction include epithelial overgrowth following childbirth, minor cervical trauma, or chronic inflammation such as cervicitis [1, 3]. Typically, Nabothian cysts are small (2–10 mm), asymptomatic, and discovered incidentally during pelvic exams or imaging studies [5]. However, in rare instances, they can grow significantly larger, beyond 4 cm, earning the designation of “giant” Nabothian cysts. These larger cysts may cause symptoms such as pelvic pressure, vaginal discharge, or, in exceptional cases, mechanical obstruction of the cervical canal [2, 4, 6, 8, 9].

The clinical significance of giant Nabothian cysts extends beyond their rarity due to their potential to mimic more sinister pathologies. On imaging, they may resemble adenoma malignum, a rare subtype of cervical adenocarcinoma with a poor prognosis, or other cystic cervical malignancies [2]. This diagnostic overlap often requires a multimodal approach, incorporating transvaginal ultrasonography, magnetic resonance imaging (MRI), and histopathological analysis to rule out malignancy [7]. While small Nabothian cysts are widely regarded as benign and inconsequential, the impact of their giant counterparts on reproductive health is less understood. Emerging literature hints at a possible link to infertility, suggesting that large or multiple cysts could obstruct sperm passage through the cervical canal or disrupt the cervical mucus environment essential for fertilization [4, 10].

These hypotheses, however, lack robust evidence and remain speculative. In the context of assisted reproductive technology (ART), the presence of giant Nabothian cysts introduces additional considerations. ART procedures, such as embryo transfer, rely on unobstructed access to the uterine cavity, and large cervical cysts could complicate catheter insertion or increase the risk of procedural infections. This case report details the management of giant Nabothian cysts in a woman undergoing ART for male factor infertility, offering insights into their clinical relevance and the feasibility of concurrent cyst aspiration during treatment.

## Case presentation

A 41-year-old Iranian woman was referred to the infertility clinic at Taleghani Hospital in Tehran with a 2-year history of primary infertility. She had been trying to conceive naturally without success and sought evaluation to identify any underlying causes. Her menstrual cycles were regular (28–30 days), and she denied experiencing symptoms such as pelvic pain, dyspareunia, or abnormal vaginal discharge. Her gynecological history was unremarkable, with no prior cervical procedures, sexually transmitted infections, or abnormal Papanicolaou (Pap) smears. She had no significant medical comorbidities, no history of smoking or alcohol use, and no family history of gynecological malignancies or infertility. During physical examination, the cervix appeared enlarged, with multiple palpable cystic structures ranging from 2 to 4 cm in diameter, completely occluding the cervical os. Bimanual palpation revealed a ballooned posterior cervix, suggesting significant cystic expansion.

**Fig. 1:**
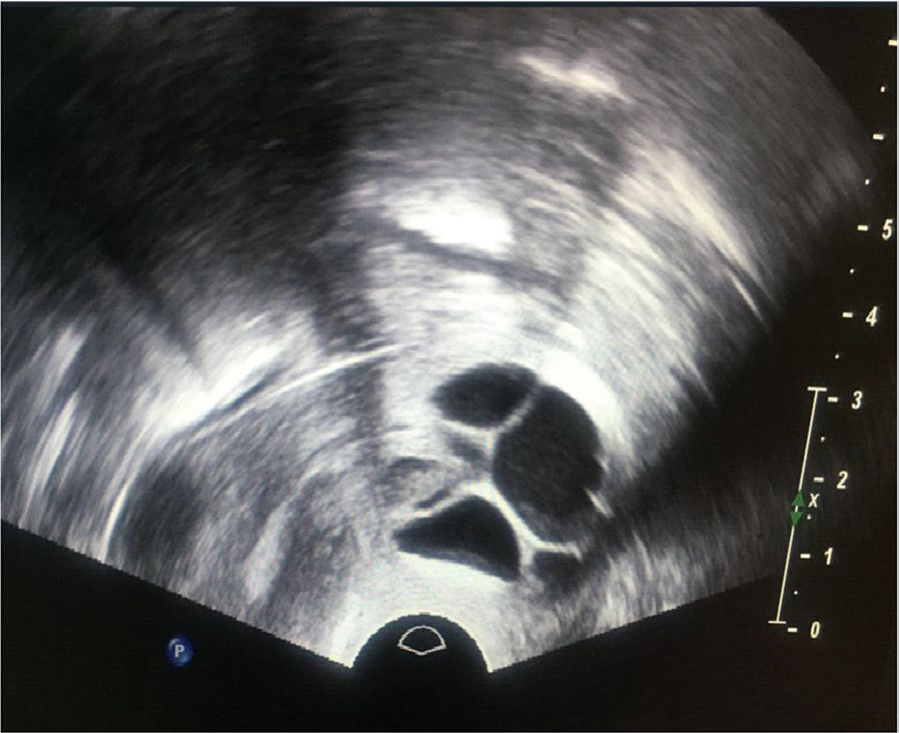
Transvaginal ultrasound: sagittal view showing anechoic cysts in the cervix

A Pap smear performed at the initial visit was negative for intraepithelial lesions or malignancy, providing reassurance but not fully addressing the nature of the cysts. Transvaginal ultrasonography was conducted as part of the infertility workup, revealing a normal uterus and ovaries but identifying multiple anechoic cervical cysts measuring 20–45 mm, protruding into the vaginal canal (Fig. 1).

**Fig. 2:**
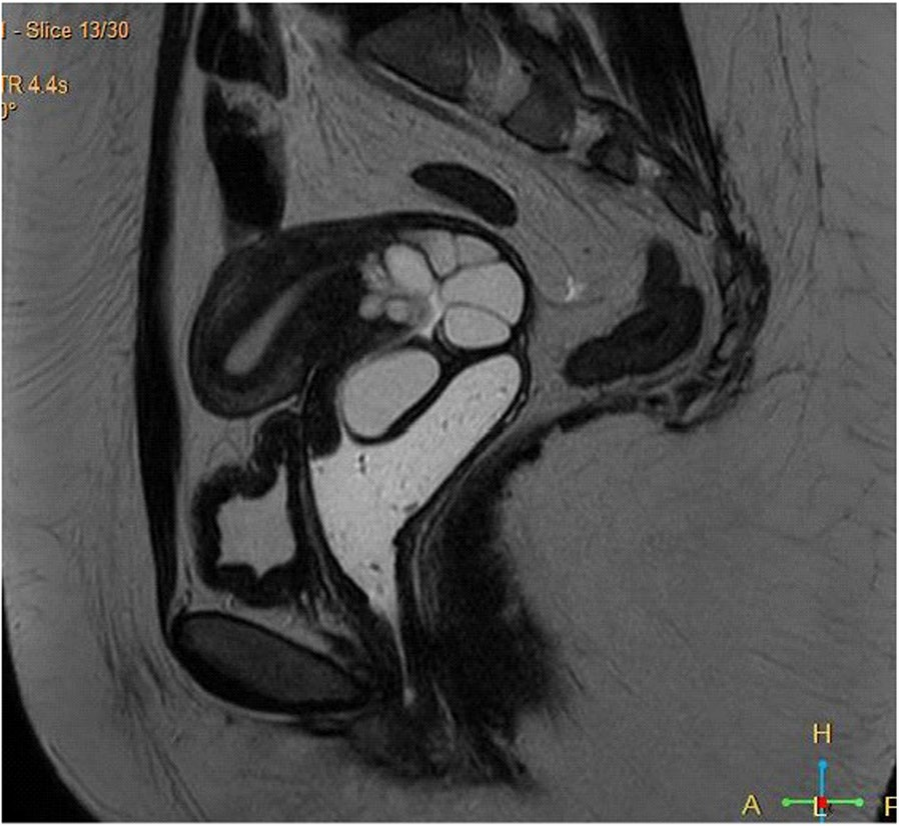
Magnetic resonance imaging: sagittal T2- weighted image showing large, hyperintense cervical cysts

The size and multiplicity of these cysts raised concerns about potential malignancy, prompting further imaging. Pelvic MRI with and without gadolinium contrast was performed, demonstrating multiple enhanced cervical lesions with characteristics suggestive of Nabothian cysts rather than malignancy (Fig. 2).

To definitively exclude adenoma malignum or other malignant entities, a cervical biopsy was obtained under colposcopic guidance. Histopathology confirmed benign Nabothian cysts, characterized by mucin-filled spaces lined by endocervical epithelium, with no evidence of atypical cells. The infertility evaluation included a comprehensive hormonal assessment, which showed follicle-stimulating hormone (FSH) at 7.5 mIU/mL, luteinizing hormone (LH) at 4.4 IU/L, estradiol (E2) at 52.6 pg/mL, anti-Müllerian hormone (AMH) at 1.2 ng/mL, and normal prolactin and thyroid-stimulating hormone (TSH) levels—indicating adequate ovarian reserve for her age. Her partner, aged 43 years, underwent semen analysis, which revealed severe teratozoospermia (only 2% normal sperm morphology), with normal sperm count and motility. This male factor infertility was deemed the primary contributor to the couple’s reproductive challenges, leading to a recommendation for intracytoplasmic sperm injection (ICSI). During the ART cycle, which commenced with an antagonist protocol, ovarian stimulation began with recombinant human FSH (Cinnal-f, Follitropin alfa, CinnaGen, Iran) at 225 IU daily for 5 days, followed by highly purified human menopausal gonadotropin (hMG; PDHMG, Pooyesh Darou, Iran) at 150 IU daily for 4 days. A gonadotropin-releasing hormone (GnRH) antagonist (Cetronax, cetrorelix acetate, Ronak Pharma, Iran) was initiated when the leading follicle reached 14 mm.

**Fig. 3:**
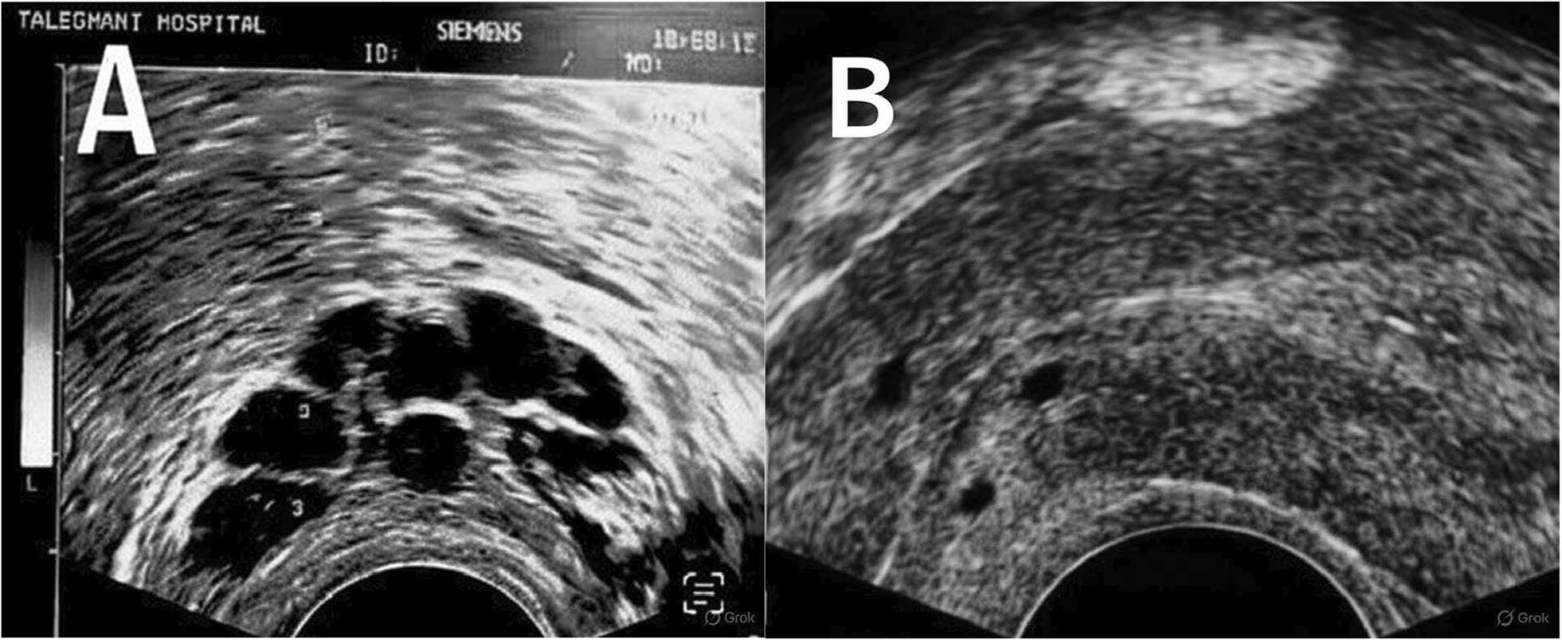
Transvaginal ultrasound images showing the cervical cysts (**A**) before and (**B**) after aspiration

After 9 days of stimulation, seven follicles exceeded 17 mm, and ovulation was triggered with 10,000 IU of human chorionic gonadotropin (hCG; PDpreg, Pooyesh Darou, Iran). Oocyte retrieval was scheduled 36 hours later. During the transvaginal oocyte retrieval, ultrasound guidance revealed seven oocytes, five of which were mature (metaphase II). However, the procedure was complicated by the presence of the large cervical cysts, which obstructed the cervical os and posed a potential barrier to subsequent embryo transfer. After discussion among the clinical team, a decision was made to aspirate the cysts concurrently using the double-lumen needle already in use for oocyte retrieval. This pragmatic approach aimed to eliminate any mechanical hindrance to catheter passage and reduce the theoretical risk of infection from the retained cystic fluid. The patient had provided prior written consent for the ART procedure, including any clinically necessary interventions, and the aspiration proceeded uneventfully. The aspirated mucoid fluid was sent for cytological analysis, which later confirmed benign endocervical cells. Post-retrieval, four of the five mature oocytes were fertilized via ICSI, yielding an 80% fertilization rate. By day 3, three embryos were classified as Grade A (high quality) and two as Grade B (fair quality). Two Grade A embryos were selected for transfer, performed transcervically without difficulty following cyst aspiration. Luteal-phase support was provided with progesterone supplementation. A serum beta-hCG test 2 weeks post-transfer was negative, indicating no pregnancy. Follow-up ultrasonography confirmed successful cyst drainage (Fig. 3), and the patient experienced no procedural complications.

## Discussion

Nabothian cysts are a common benign finding in reproductive-aged women, but their presentation as giant cysts is exceptionally rare. In this case, the cysts’ size (20–45 mm) and obstruction of the cervical os necessitated a detailed diagnostic process to differentiate them from malignancy. The combination of transvaginal ultrasonography, MRI, and biopsy provided a robust confirmation of their benign nature, aligning with best practices for managing atypical cervical lesions [2, 7]. This thorough evaluation was critical, as misdiagnosis could have delayed fertility treatment or led to unnecessary surgical intervention.

The couple’s infertility was primarily attributed to severe male factor infertility (teratozoospermia), with the patient’s ovarian reserve and uterine anatomy appearing normal. However, the discovery of giant Nabothian cysts raised questions about their potential contribution to the couple’s reproductive difficulties. Although the literature on Nabothian cysts and infertility is limited, some studies suggest that large cysts might impede fertility by obstructing sperm transport or altering cervical mucus composition [4, 10]. In natural conception, such mechanisms could theoretically play a role, but in ART, where sperm bypasses the cervix via ICSI, these effects are less relevant. Instead, the cysts’ significance in this case lay in their potential to complicate procedural aspects of ART, such as embryo transfer.

The decision to aspirate the cysts during oocyte retrieval was a proactive measure to optimize the ART cycle. By removing the cysts, we aimed to ensure unobstructed catheter access and minimize infection risk, considerations particularly relevant given the patient’s advanced reproductive age and the limited window for successful treatment. The procedure’s success without additional morbidity supports the feasibility of this approach in similar scenarios. However, the cycle’s failure to achieve pregnancy underscores the multifactorial nature of infertility, where addressing one variable (e.g., cervical cysts) may not overcome others, such as embryo quality or endometrial receptivity.

The broader implications of this case highlight gaps in the current understanding of Nabothian cysts’ reproductive impact [12]. While anecdotal reports describe spontaneous pregnancies following cyst removal [3], and cross-sectional studies note associations with infertility [10, 11], causal relationships remain unproven. In ART, where technical precision is paramount, managing such cysts may enhance procedural success, but their direct impact on female fertility and pregnancy outcomes remains unclear and requires further investigation. Future research should focus on prospective studies to assess the prevalence and effects of giant Nabothian cysts in infertile populations, particularly those undergoing ART.

In summary, we would like to emphasize again that the primary reason for utilizing ART through ICSI was the presence of severe male factor infertility, specifically teratozoospermia. Additionally, the aspiration of the large Nabothian cysts was performed to optimize the ART process by minimizing the risk of potential procedural complications. It was not intended as a direct treatment for the couple’s infertility.

It can be generally stated that, in addition to the potential for physical obstruction, large Nabothian cysts might theoretically affect fertility through various mechanisms, such as changes in cervical mucus secretion or the promotion of chronic inflammation, according to existing literature [10, 11]. However, in the context of this specific case, the relevance of the cysts was predominantly procedural rather than etiological.

## Conclusion

This case report details the successful management of giant Nabothian cysts via aspiration during an ART cycle in a woman with primary infertility due to male factor issues. The intervention was technically feasible, safe, and potentially beneficial in facilitating embryo transfer, yet it did not result in pregnancy, emphasizing the complexity of infertility treatment. Clinicians should consider the presence of large cervical cysts in ART planning, tailoring interventions to individual patient needs. Additional research is essential to clarify the clinical significance of Nabothian cysts in fertility and to guide their management in reproductive medicine.

## Data Availability

Data are available from the corresponding author upon reasonable request.
